# Low-Dose Naltrexone in Rheumatological Diseases

**DOI:** 10.31138/mjr.34.1.1

**Published:** 2023-03-31

**Authors:** Jozélio Freire de Carvalho, Thelma Skare

**Affiliations:** 1 Núcleo de Pesquisa em Doenças Crônicas não Transmissíveis (NUPEN), School of Nutrition from the Federal University of Bahia, Salvador, Bahia, Brazil,; 2 Unit of Rheumatology, Hospital Evangélico Mackenzie, Curitiba, PR, Brazil

**Keywords:** naltrexone, low-dose naltrexone, opioid system, rheumatic diseases, rheumatoid arthritis, dermatomyositis, fibromyalgia, Sjögren’s syndrome

## Abstract

**Background:**

Naltrexone has been approved for alcohol and opioid abuse by the FDA. At low-dose naltrexone (LDN) has been used in several diseases including chronic pain and autoimmune conditions, including rheumatic disorders.

**Aim:**

To review the use of LDN in rheumatic diseases: systemic sclerosis (SSc), dermatomyositis (DM), Sjögren’s syndrome (SS), rheumatoid arthritis (RA), and fibromyalgia (FM).

**Methods:**

PubMed and Embase databases were searched for articles on LDN and rheumatic diseases between 1966 and August 2022.

**Results:**

Seven studies in FM have been identified: in this disease LDN has showed beneficial effects on pain and well-being. In SS, two articles with 3 cases description showed that LDN may be of help in the pain treatment. LDN relieved pruritus in scleroderma (a case description with a series of 3 patients) and dermatomyositis (description of 3 patients in two articles). In RA a study using Norwegian Prescription Database showed that LDN was associated to reduction in the use of analgesic and DMARDs. No serious side effects were detected.

**Conclusion:**

This review shows that LDN is a promising and safe therapy to be used in some rheumatic disease. However, the data is limited and needs to be reproduced in larger studies.

## INTRODUCTION

Low dose naltrexone (LDN) has been proposed as a new form of analgesic and anti-inflammatory treatment for several chronic pain conditions.^1^ This compound is functionally and structurally similar to the opioid antagonist naloxone, but with longer half-life and better oral bioavailability, and it is usually prescribed for treatment of opioid addiction.^2^ The typical dosage of naltrexone used for treatment of opioid addiction ranges from 50 to 100mg/day^1^; LDN refers to a dosage of 1–6 mg/day.^2^ At such low levels, naltrexone exhibits paradoxical anti-inflammatory and analgesic properties. The analgesic effect of LDN results from the blockage of mu- and delta-opioid receptors and to a lesser extent kappa-opioid receptors in the central nervous system leading to a feedback-mediated increase of these receptors and improving the endorphin system.^1,3,4^ The anti-inflammatory effects are due to blockage of the toll like receptor 4 (TLR-4) in the microglia cells, at central nervous system.^5^ These microglia cells, when chronically stimulated produce several pro-inflammatory cytokines, substance P, nitric oxide, and excitatory amino acids that are associated to the so-called sickness behaviour: cognitive impairment, mood and sleep disorders, fatigue, pain amplification and malaise, a group of symptoms similar to those found in patients with fibromyalgia (FM).^1^

LDN has been used as an alternative for treatment of several rheumatologic conditions such as FM, dermatomyositis and Sjögren’s syndrome.^6–19^ Herein a systematic review of LDN safety and efficacy LDN in rheumatological conditions is performed.

## METHODS

Literature review: A systematic search of articles published in PubMed/MEDLINE, EMBASE and Scielo from 1966 to August 2022 using the following MeSH entry terms: “low-dose naltrexone” OR “naltrexon” and “rheumatic” OR “rheumatologic” OR “systemic lupus erythematosus” OR “lupus” OR “fibromyalgia” OR “rheumatoid arthritis” OR “spondyloarthritis” OR “Sjögren’s syndrome” OR “myositis” OR “systemic sclerosis” OR “vasculitis” OR “Takayasu disease” OR “Wegener’s disease” OR “granulomatosis with polyangiitis” OR “Kawasaki’s disease” OR “polyarteritis nodosa” OR “Livedoid vasculitis” OR Churg-Strauss” OR “eosinophilic granulomatosis with polyangiitis” OR “osteoarthritis” OR “gout.”. The search had no language restriction. The reference lists of the selected articles were analysed to identify other publications.

Initially, two authors (JFC and TLS) performed the literature search and independently selected the study abstracts. In the second stage, the same reviewers independently read the full-text articles selected by abstracts. The authors followed PRISMA guidelines.^20^ A standardized form to extract the information from relevant articles was design including authors, year of publication, number of patients studied, demographic data, disease duration, study follow-up, LDN posology, outcomes, and side effects.

## RESULTS

**Figure 1.**
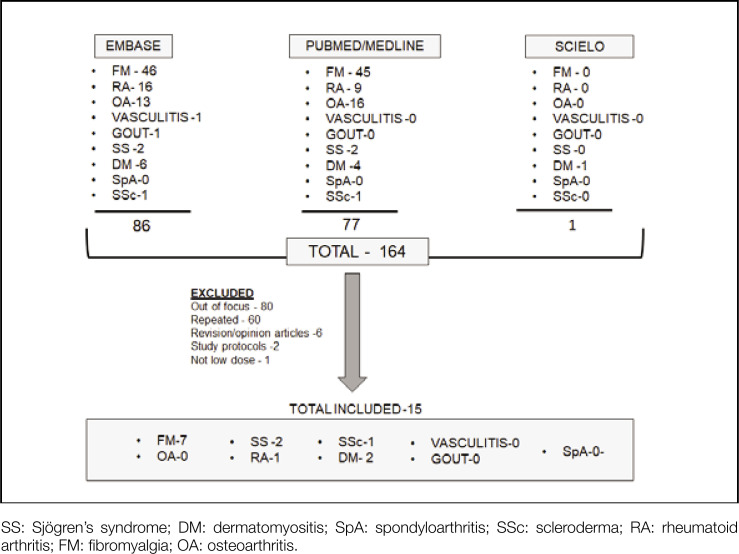
Flowchart of the studies on LDN and rheumatic diseases.

**Figure 1** shows the flowchart with search results. In this context, FM was the disease most studied.

**Table 1** summarises the search results on LDN treatment in fibromyalgia. In the seven reviewed studies, six small prospective trials and one case description were found. They included mostly females (90.5 to 100%) using 4,5 mg naltrexone/day, encompassing a total of 121 treated adult individuals. Regarding outcome, all studies showed good results after LDN treatment, with improvement of pain, FM scales and inflammatory parameters such as cytokines. Most of the studies revealed no side effects or showed mild effects such as insomnia or vivid dreams.

**Table 1.** Studies of low dose naltrexone in fibromyalgia.

**Author, reference**	**Study design**	**N,**	**Age/gender**	**Disease duration**	**LDN dose (mg/day)**	**Outcome**	**Side effects**
Siembida & Johnson, 2022^8^	Case report	1	60/male	8 years	0.2 → 9mg	Remission of fibromyalgia and depression. ✓ HAM-D from 20 →0; ✓ CPT from 21sec →26sec; ✓ FPS from 5/10 → 0/10	None
Jackson et al., 2021^10^	7-weeks, open label	21	43.4y; 90.5% females	NA	0.1 → 4.5mg/twice daily	✓ CPT from 14s→30s ✓ increased 0/10(2x) pain tolerance.	None
Bruun-Plesner et al., 2020^13^	Single-blinded clinical trial using “up-and-down” method – 3 weeks.	25	47.0 ± 9.4 y; 100 % females	13.6±11.1 years	0.75 → 6mg	✓ Improvement of FIQR (−13.6 ±12.1); ✓ Improvement of pain (−0.4 ±2.0) ✓ Improvement of ISI (−5.4 ±4.6) ✓ ED50 estimated in 3.88 mg ✓ ED95 estimated in 5.40 mg.	Mild gastrointestinal symptoms (abdominal pain; diarrhoea)
Parkitny et al., 2017^7^	10-weeks, single-blind, crossover trial	8	46 y; 100% females	14 years	4.5mg	✓ 15% reduction of pain; ✓ 18% reduction overall symptoms; ✓ Reduced plasma concentrations of IL-1B, 1-Ra, IL-2, IL-4, IL5, IL-6, IL-10, IL-12p70, IL-15, IL-17A, IL-27, INF-α, TGF-α and β and G-CSF.	None
Metyas et al., 2018^9^	Open, prospective	25	30–75 y; 96% females	NA	1.5 → 4.5 mg	Reduced FIQR in 90 days	NA
Younger et al., 2009^6^	Single-blind, crossover trial – 14 weeks	10	44.0 ± 10 y; 100% females.	9.6 ± 6.5 years	4,5 mg	✓ Reduced 30% symptoms severity; ✓ Mechanical pain thresholds raised by 0.22 k; ✓ Thermal pain thresholds were increased by 0.9°C. Baseline ESR predicted over 80% of the variance in drug response.	Minor: insomnia and vivid dreams
Younger et al., 2013^12^	Randomized, double-blind, placebo-controlled, counterbalanced, crossover trial; 20 weeks.	31,	42.7 ± 12.9 yo, 100% females	11.7 ±10.1 years	4.5 mg	28.8% reduction in baseline pain (vs 18.0% of controls) Improvement of mood and general satisfaction with life; No improvement of fatigue and sleep.	Equal to placebo.

IL: interleukin; INF: interferon; TGF: transforming growth factor; G-CSF: granulocyte-colony stimulating factor; ESR: erythrocyte sedimentation rate; HAM-D: Hamilton depression rating scale; CPT: cold pressor test; FPS, face pain scale: FIQ-R-fibromyalgia impact questionnaire-revised; ISI: insomnia severity index; ED50: dose effective in 50% of subjects; ED95: effective dose in 95% of subjects; NA: not available.

**Table 2** shows the results of the search for the other rheumatic diseases: two case description in Sjögren’s syndrome (including 3 patients) as well as two cases description in dermatomyositis (also with 3 patients). In rheumatoid arthritis (RA), a controlled before-after study with 360 patients with seropositive disease and in scleroderma, a series of case description including 3 patients were identified. In these studies, females predominated (from 50% to 100%); age varied from 67 to 66 years old, and disease duration from 1 to 6 years. LDN dosage varied from 2.0 to 8.5 mg/day. Regarding outcome, all of them showed a good result after LDN treatment, with improvement of pain, pruritus) (in dermatomyositis and scleroderma) and gastrointestinal symptoms (in scleroderma). In 7/8 of the studies no side effects were observed, while in one study, no description was found. (**Table 2**).

**Table 2.** Studies in low dose naltrexone use in Sjögren’s syndrome, dermatomyositis, systemic sclerosis, and rheumatoid arthritis.

**Author, reference**	**Study design**	**N, age, gender**	**Rheumatic disease**	**Disease duration**	**LDN dose, initial → final (mg/day)**	**Outcome**	**Side effects**
Zashin S, 2019^15^	Case report	N=1 47 y, female	Sjögren’s syndrome	6 years	1.5 → 4.0	Good. Improved fatigue, pain, ESR and CRP. No changes in dryness (eye and mouth).	None
Zashin S, 2020^14^	Case report	N=2 Case 1: 66 y, female Case 2: 24 y, female	Sjögren’s syndrome	Case 1: ND Case 2: 5 years	Case 1: 1.0 → 2.0 Case 2: 0.5 → 8.5	Good. Case 1: Improved pain, ESR and CRP. Case 2: Reduced arthralgia, headache, CRP and ESR.	None
Tran et al., 2018^16^	Case report	N=2, Case 1: 34 y, male; Case 2: 54 y, female	Dermato myositis	Case 1: 2 years Case 2: NA	5 mg	Case 1: Improved skin lesions, pruritus, arthralgia, and muscle pain Case 2: Improved skin rash and pruritus.	NA
Manudhane et al, 2019^17^	Case report	N=1, 56y, male	Amyopathic dermatomyositis	NA	1.5 → 4.5 mg	Improved pruritus, burning sensation, body, and facial rash.	None
Frech et al., 2011^18^	Case report	N=3, 34 to 56 y; 100% female	Systemic sclerosis (2 diffuse and 1 limited)	1 to 2 years	2.0 → 4.5mg	A trend towards improvement of modified Rodnan skin score, Improvement of GIT score, pruritus, and constipation.	None
Raknes et al., 2019^19^	Controlled before-after study	N=360, 58.7 to 60 y, 76.9% females	Rheumatoid arthritis and seropositive arthritis	NA	>5mg/day	For persistent LDN users, reduction in 13% of rheumatic drugs (NSAID, opioids, DMARDs and TNF blockers), and analgesics was observed	NA

N: number; NA: not available; ESR: erythrocyte sedimentation rate; CRP: C-reactive protein; LDN: low dose naltrexone; NSAID: nonsteroidal anti-inflammatory drugs; DMARDs: disease-modifying antirheumatic drugs; TNF: tumoral necrosis factor; GIT: gastrointestinal tract.

## DISCUSSION

This is the first study to systematically review the therapeutic effects of LDN in rheumatic diseases. Although the use of LDN in this context is still off-label, the studies presently reviewed show that this form of treatment should be explored to benefit patients with refractory FM and refractory pruritus in cases of dermatomyositis and scleroderma.^6,12,18^ In FM it seems not just to alleviate pain, but also to improve overall symptoms, probably because of its anti-inflammatory properties at SNC levels, inhibiting the production of several cytokines locally.^12^ A systemic anti-inflammatory effect cannot be ruled out as the work by Younger et al.^12^ shows that LDN therapeutic response is proportional to patient’s baseline ESR and the work by Parkitny et al.^7^ demonstrated that the levels of several pro inflammatory cytokines are decreased in the periphery after its administration. Furthermore, animal studies have shown that naloxone suppresses the production of IL-6, TNF-alpha, monocyte chemoattractant protein-1, and superoxide in peripheral macrophages.^21^ This is a fascinating aspect of LDN, once FM does not respond to common anti-inflammatory drugs such as AINHs or glucocorticoids and cannot be considered an inflammatory disorder from the classic point of view.^22,23^ Even though some degree of inflammation may exist at SNC level, in microglia cells; the cytokine induced sickness behaviour that results from microglia inflammation overlaps many symptoms with FM.^1^

Some possible explanations for the anti-inflammatory and immune regulatory properties of LDN might be that this drug could (1) regulate T lymphocyte subsets: CD4+/CD8+T cells, Th1/Th2 cells and Th17/Treg cells; (2) decrease the TNF-α, IL-6, IL-12 alpha and IL-17 expression, (3) increase the expression of IL-10, an anti-inflammatory cytokine, and (4) regulate immune responses to reconstruct the immune balance to alleviate inflammation.^24^

Another point that needs to be explored is the LDN action in pruritus with beneficial aspects in dermatomyositis and scleroderma.^16–19^ Pruritus is a symptom that can be associated with significant morbidity and loss of quality of life and that has restricted treatment options.^18^ The pathophysiology of this symptoms is not completely understood but it may involve an amplified opioid-mediated neurotransmission in the brain.^25^ Therefore, LDN may control pruritus through opioid mediated actions or decreasing inflammatory mediators.^18^ Interestingly, in scleroderma, pruritus has been linked to gastrointestinal symptoms that also improved with LDN.^18^

Finally, a controlled before-after study grounded on the Norwegian Prescription Database (NorPD) compared prescriptions to RA patients one year prior to and one year after starting LDN and found a reduction in the use of NSAID, opioids, and DMARDs such as methotrexate and anti TNF-alpha drugs.^19^ Additionally to its analgesic and anti-inflammatory properties, LDN may work as an immunomodulating agent by directly binding on the opioid growth factor receptor (OGFr) within immune and tumour cells.^26^

Advantages of this form of treatment are that LDN is affordable, and it has a low incidence of adverse side effects^1^ being considered safe at low dosage.^17^

Some limitations were observed in this study. For instance, no comparison between LDN and classical treatments used in rheumatic diseases were available. The number of participants was low, and the follow-up was short. Future studies should include larger patient samples with more long-term observation; this would enable a better understanding of the course of LDN in rheumatic conditions. The study strengths are: (1) inclusion of studies with patients with international criteria for rheumatic diseases; and (2) inclusion of all kinds of study design of the use of LDN in rheumatic diseases. In this way, the authors believe that all published cases of LDN in rheumatic patients were collected.

## CONCLUSION

There are few articles in the literature evaluating LDN in rheumatological diseases, and only five such diseases were addressed in this review. All studies analysed demonstrated that the LDN use seems to be efficacious in treating signs and symptoms of rheumatic diseases (pain, FM scales and cytokine levels) and as well as with a few rare and minor side effects. So LDN emerges as an interesting option to be explored in the rheumatological field.
